# Immunogenicity and Protective Efficacy of the DAR-901 Booster Vaccine in a Murine Model of Tuberculosis

**DOI:** 10.1371/journal.pone.0168521

**Published:** 2016-12-20

**Authors:** Timothy Lahey, Dominick Laddy, Krystal Hill, Jacqueline Schaeffer, Alison Hogg, James Keeble, Belinda Dagg, Mei Mei Ho, Robert D. Arbeit, C. Fordham von Reyn

**Affiliations:** 1 Dartmouth’s Geisel School of Medicine, 1 Medical Center Drive, Lebanon, NH, United States of America; 2 Aeras, 1405 Research Blvd. Rockville, MD United States of America; 3 Bacteriology Division, MHRA-NIBSC, South Mimms, Potters Bar, Hertfordshire, United Kingdom; 4 Tufts University School of Medicine, Boston, MA United States of America; University of Cape Town, SOUTH AFRICA

## Abstract

**Background:**

The development of a novel tuberculosis vaccine is a leading global health priority. SRL172, an inactivated, whole-cell mycobacterial vaccine, was safe, immunogenic and reduced the incidence of culture-confirmed tuberculosis in a phase III trial in HIV-infected and BCG immunized adults in Tanzania. Here we describe the immunogenicity and protective efficacy of DAR-901, a booster vaccine against tuberculosis manufactured from the same seed strain using a new scalable method.

**Methods:**

We evaluated IFN-γ responses by ELISpot and antibody responses by enzyme linked immunosorbent assay in C57BL/6 and BALB/c mice after three doses of DAR-901. In an aerosol challenge model, we evaluated the protective efficacy of the DAR-901 booster in C57BL/6 mice primed with BCG and boosted with two doses of DAR-901 at 4 dosage levels in comparison with homologous BCG boost.

**Results:**

DAR-901 vaccination elicited IFN-γ responses to mycobacterial antigen preparations derived from both DAR-901 and *Mycobacterium tuberculosis*. DAR-901 immunization enhanced antibody responses to DAR-901 but not *Mycobacterium tuberculosis* lysate or purified protein derivative. Among animals primed with BCG, boosting with DAR-901 at 1 mg provided greater protection against aerosol challenge than a homologous BCG boost (lungs P = 0.036, spleen P = 0.028).

**Conclusions:**

DAR-901 induces cellular and humoral immunity and boosts protection from *M*. *tuberculosis* compared to a homologous BCG boost.

## Introduction

Tuberculosis sickens over eight million people and kills more than one million every year [1, 2]. The standard vaccine against tuberculosis, *Mycobacterium bovis*, bacille Calmette-Guerin (BCG), is effective when administered to mycobacteria-naïve infants, but protection appears to wane after 10–15 years [3] and boosting with another administration of BCG is ineffective [4, 5] The development of improved vaccines against tuberculosis is thus a critical global health priority [6]. Modeling studies indicate that an effective BCG booster for adolescents and adults will have a greater impact on the global pandemic than an improved BCG for priming infants [7].

Our work has focused on development of a booster vaccine for BCG. We have shown previously that a multiple-dose series of SRL172, an inactivated, whole-cell vaccine prepared from a non-tuberculous mycobacterium was safe, well-tolerated and immunogenic in humans [8–10]. A randomized controlled Phase III trial in Tanzania demonstrated that boosting with SRL172 protected against culture-confirmed tuberculosis in HIV-infected adults who had received BCG at birth [11].

DAR-901, a vaccine produced from the same seed strain using a new, scalable manufacturing method, has entered clinical development. In this report, we present the results of preclinical studies of the immunogenicity and protective efficacy of DAR-901 in mice.

## Methods

**Fig 1:**
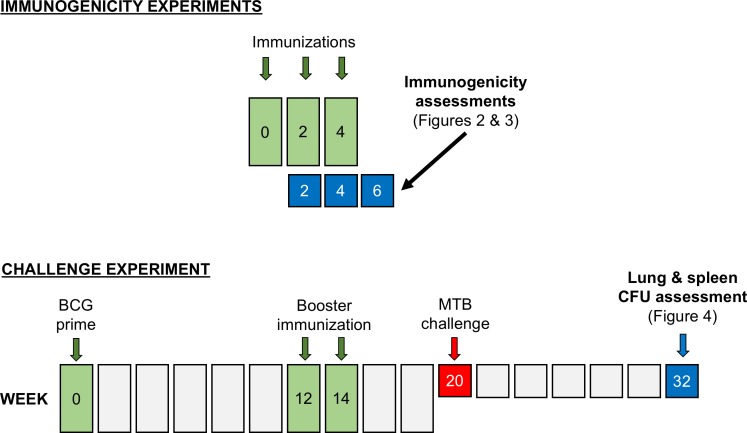
Schedule of study visits and assessments. In immunogenicity experiments, mice were immunized with varying doses of DAR-901 at weeks 0, 2 and 4, and assessments of antigen-specific interferon gamma responses were undertaken at weeks 2, 4 and 6. Antibody responses were assessed at week 2. In our challenge experiment, mice were immunized with BCG vaccine at week 0, and then boosted with the indicated saline, BCG or DAR-901 dose levels at weeks 12 and 14. At week 20 mice were challenged with *Mycobacterium tuberculosis* (MTB). For mice in challenge experiments, growth of MTB was assessed in lungs and spleen at week 32. BCG, bacille Calmette Guerin; CFU, colony forming units; MTB, *Mycobacterium tuberculosis*

Fig 1 depicts a schedule of study visits and assessments.

### Mice

#### Immunogenicity Experiments

We obtained female 8–10 week old C57BL/6 and BALB/c mice from Jackson Laboratory (Bar Harbor, Maine) and maintained them in pathogen-free conditions. Animal experiments were reviewed and approved by the Noble Life Sciences Institutional Animal Care and Use Committee (IACUC). We conducted experiments according to guidelines set out by the Association for Assessment and Accreditation of Laboratory Animal Care International (AAALAC). Prior to vaccine administration at weeks 0, 2 and 4, mice were anesthetized using tribromoethanol 250mg/kg intraperitoneally. Immunogenicity assessments were performed in mice sacrificed by carbon dioxide asphyxiation at weeks 2, 4 and 6. Animals were monitored continuously throughout experimental procedures, routinely following any procedures (e.g., to ensure full recovery from anesthesia), and otherwise on a daily basis. No procedures involved in this study caused more than momentary pain/distress (e.g., subcutaneous immunizations). Our IACUC protocol allows the vivarium to euthanize any animals that appear to be in any pain or distress. No animals died prior to the experimental endpoint; there were no observations of significant pain/distress.

#### Challenge experiments

We obtained specific pathogen-free, female 6–9 weeks old C57BL/6 mice from Charles River (United Kingdom). All animal procedures were approved by the National Institute for Biological Standards and Control ethics committee and were in accordance with UK Home Office (Scientific Procedures) Act 1986. Mice were primed with BCG at week zero, boosted with the indicated immunization at weeks 12 and 14, challenged by exposure to *Mycobacterium tuberculosis* at week 20, and growth of *M*. *tuberculosis* in spleen and lung assessed at week 23 (as below) following euthanasia by cervical dislocation. Throughout the study, mice were monitored by trained animal technicians at least once a day. If any adverse reactions or abnormalities were observed, the monitoring frequency could be increased. Per protocol, mice with greater than 20% loss of body weight and mice with adverse reactions that exceeded the “Moderate” severity grade were to be sacrificed.

### Vaccine

#### Vaccine preparation

DAR-901 is an inactivated, whole cell mycobacterial vaccine prepared from the Master Cell Bank for SRL172, which was prepared from organisms grown on agar. DAR-901 was manufactured by Aeras (Rockville, MD) using organisms grown by broth fermentation. SRL172 was originally identified as a *Mycobacterium vaccae* based on phenotypic characteristics [12]. 16S rRNA gene sequencing of the seed strain demonstrates >99.6% identity to the reference 16S rRNA sequence for *Mycobacterium obuense* and 100% identity to SRL172. DAR-901 is distributed in 2 mL vials containing 0.3–0.4 mL of a 1 mg/mL suspension of heat-inactivated organisms.

#### Vaccination procedure

Intradermal immunizations were performed using insulin syringes (BD Biosciences, San Jose, CA). DAR-901 was diluted to various doses (0.1 to 2.5 mg/dose) in citrate buffer and administered in a total volume of 50 μL. Vaccine was administered at alternating sites on the stomach of anesthetized mice every two weeks up to a total of three doses. For *in vitro* assays, mice were sacrificed at 2 weeks after the first, second and third doses, blood samples were obtained and splenocytes isolated.

### Immunological assays

#### ELISpot assay

ELISpot assays were conducted in 96-well polyvinylidene fluoride ELISpot plates (Millipore, Billerica, MA) with R&D Systems Mouse Interferon- ELISpot kit (R&D System, Minneapolis, MN), as previously described [13]. Briefly, plates were coated with anti-mouse capture antibodies and incubated overnight at 4°C. The following day, plates were washed with PBS and blocked for two hours with RPMI 1640 medium containing 10% fetal bovine serum, 55 μM mercaptoethanol (Sigma, St. Louis, MO), 10 mM HEPES buffer (Gibco, Grand Island, NY), 2 mM L-glutamine (Gibco) and 1x penicillin/streptomycin (Life Technologies) (R10). Splenocytes (2x10^5^ cells per well, in triplicate) were incubated overnight at 37°C with R10 (negative control), and 0.4 μg of each antigen.

#### Antibody ELISA

96-well Thermo Scientific Immuno Plates (Fisher Scientific, Pittsburgh, PA) were coated overnight with 1 μg/well antigen at 2–8°C, washed with 1X PBS containing 0.05% Tween-20, blocked with 1X PBS containing 5% non-fat dry milk, incubated at room temperature for two hours with diluted mouse serum, washed, incubated at room temperature for one hour with anti-mouse IgG-HRP (Santa Cruz Biotechnology, Dallas, TX), washed, incubated in the dark at room temperature with 3,3',5,5'-tetramethylbenzidine (TMB) substrate (BD Biosciences), and substrate development stopped after 30 minutes. The plates were read on a SpectraMax Plus 384 plate reader (Molecular Devices, Sunnyvale, CA). The results shown represent the mean of duplicate wells.

### Challenge study

#### Challenge

Groups of ten mice were primed intradermally with 1 × 10^5^ colony forming units (CFU) of culture grown BCG-TICE strain or with sterile saline. BCG vaccinated groups of mice were scheduled to be boosted with either three intradermal doses of DAR-901 at various dosage (0.1 to 2.5 mg/ dose) at week 12, 14 and 16, or a second dose of BCG at week 12. No anesthetics or analgesics were used at the time of injection. Six weeks after the second DAR-901 boost vaccination, five animals per group were challenged with *M*. *tuberculosis* H37Rv strain (MTb) by the aerosol route using a Glas-Col inhalation chamber, with a target dose of 50–100 CFU per lung per mouse. The remaining animals were sacrificed and used for a separate study.

#### Infection assessment

At 12 weeks post-challenge, all mice were sacrificed and bacterial load in lungs and spleens determined. Samples of each tissue were homogenised separately in cold saline + 0.01% (v/v) Digitonin with glass beads (2.5–3.5 mm), using a Fast-Prep FP120 ribolyzer at speed 6 for 30 seconds, serially diluted, plated in duplicate onto Middlebrook 7H11 agar plates supplemented with 10% oleic acid-albumin-dextrose-catalase (OADC; Difco), 0.5% glycerol, and the plates incubated at 37°C until CFU could be counted, typically in 2–3 weeks.

### Statistical analysis

We compared interferon gamma responses and post-challenge lung and spleen CFU of MTb between DAR-901 vaccine dose conditions vs saline and other comparator conditions using grouped one-way ANOVA. For grouped comparisons yielding P<0.0001 we reported the results of comparisons between individual conditions (for instance BCG plus DAR-901 1.0 mg vs BCG alone) using student’s t tests with a p value of <0.05 as the threshold for statistical significance. In comparisons of antibody responses to mycobacterial antigens, there were only single averaged values for each experimental condition so we compared the average antibody concentration of all mice that received saline to the average antibody concentration measured in all mice that received any dose of DAR-901 using a single students t test. We depicted and analyzed data in Prism 6 (GraphPad La Jolla, CA) and STATA 12 (College Station, TX).

## Results

### ELISpot

**Fig 2:**
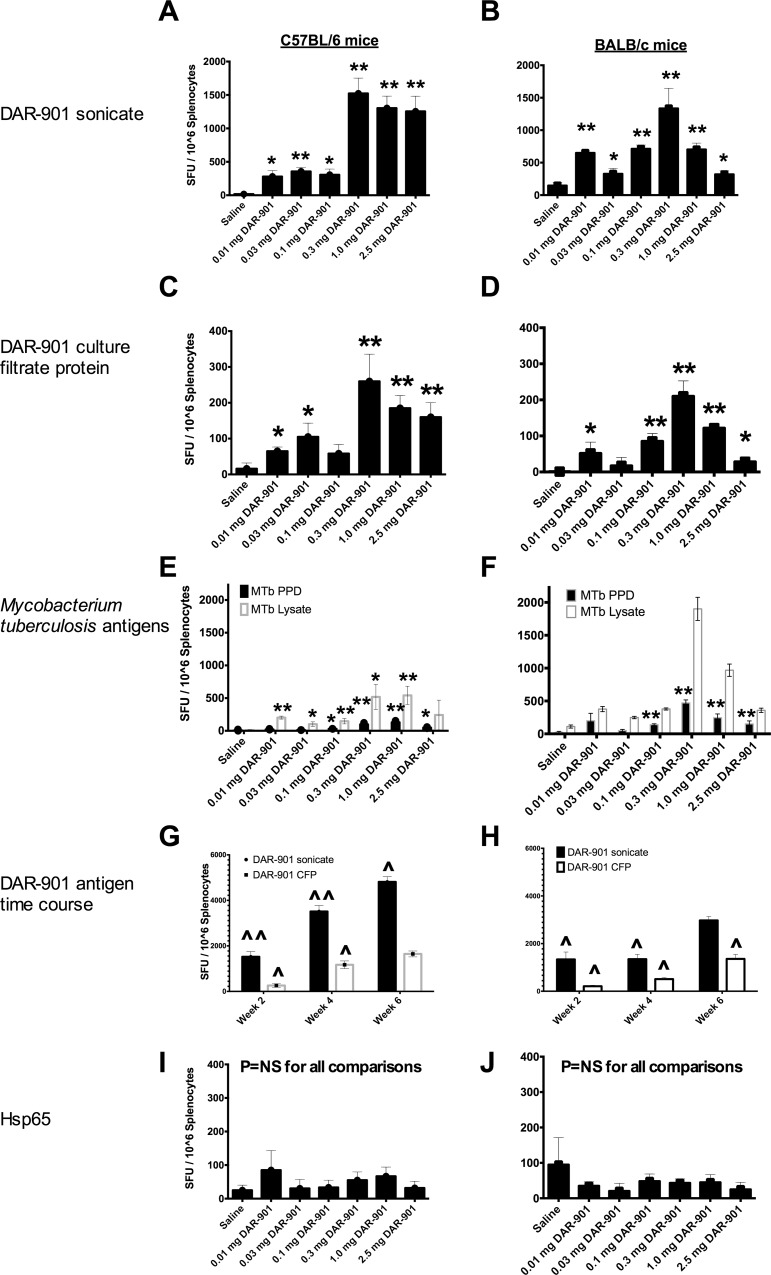
DAR-901 vaccination elicits interferon gamma (IFN-γ) responses to multiple mycobacterial antigens by ELISpot assay. Mice were immunized at week 0, 2 and 4 with the indicated dose of DAR-901. At week 2, DAR-901 vaccination elicited IFN-γ responses to DAR-901 sonicate in C57BL/6 (A) and BALB/c (B) mice, DAR-901 culture filtrate protein in C57BL/6 (C) and BALB/c (D) mice, and to *Mycobacterium tuberculosis* purified protein derivative and lysate in C57BL/6 (E) and BALB/c (F) mice. In time course experiments, IFN-γ responses to the vaccine antigens DAR-901 sonicate and DAR-901 culture filtrate protein progressively increased from 2 to 6 weeks after additional vaccinations with DAR-901 0.3 mg in C57BL/6 (G) and BALB/c (H) mice. DAR-901 vaccination did not enhance IFN-γ responses to Hsp65 in C57BL/6 (I) or BALB/c (J) mice. Splenocytes from 5 mice/dose level/time point were pooled, and the bars indicate the mean and standard deviation of triplicate repeat measurements. P values reflect the comparison of the indicated vaccine dose to the saline control condition using a student’s t test. * P value for DAR-901 response compared to saline <0.05. ** P value for DAR-901 response compared to saline < 0.01. ^ P value for DAR-901 response compared to the previous visit <0.05. ^^ P value for DAR-901 response compared to the previous visit <0.01. CFP, culture filtrate protein; MTb, *Mycobacterium tuberculosis*; PPD, purified protein derivative; SFU, spot forming units

At 2 weeks after a three-dose series, DAR-901 vaccination significantly enhanced IFN-γ responses to DAR-901 sonicate (Fig 2A and 2B), and DAR-901 culture filtrate protein (Fig 2C and 2D) compared to saline control in both C57BL/6 and BALB/c mice. In addition, DAR-901 IFN-γ responses to *M*. *tuberculosis* purified protein derivative (PPD) and lysate were significantly enhanced by DAR-901 vaccination compared to saline (Fig 2E and 2F). In time course experiments in recipients of the 0.03 mg dose of DAR-901, DAR-901-specific IFN-γ responses increased from week 2 to weeks 4 and 6 in C57BL/6 and BALB/c mice (Fig 2G and 2H, respectively). DAR-901 vaccination did not elicit IFN-γ responses to commercially available potentially cross-reactive mycobacterial heat shock protein Hsp65 (Fig 2I and 2J, respectively).

### Antibody ELISA

**Fig 3:**
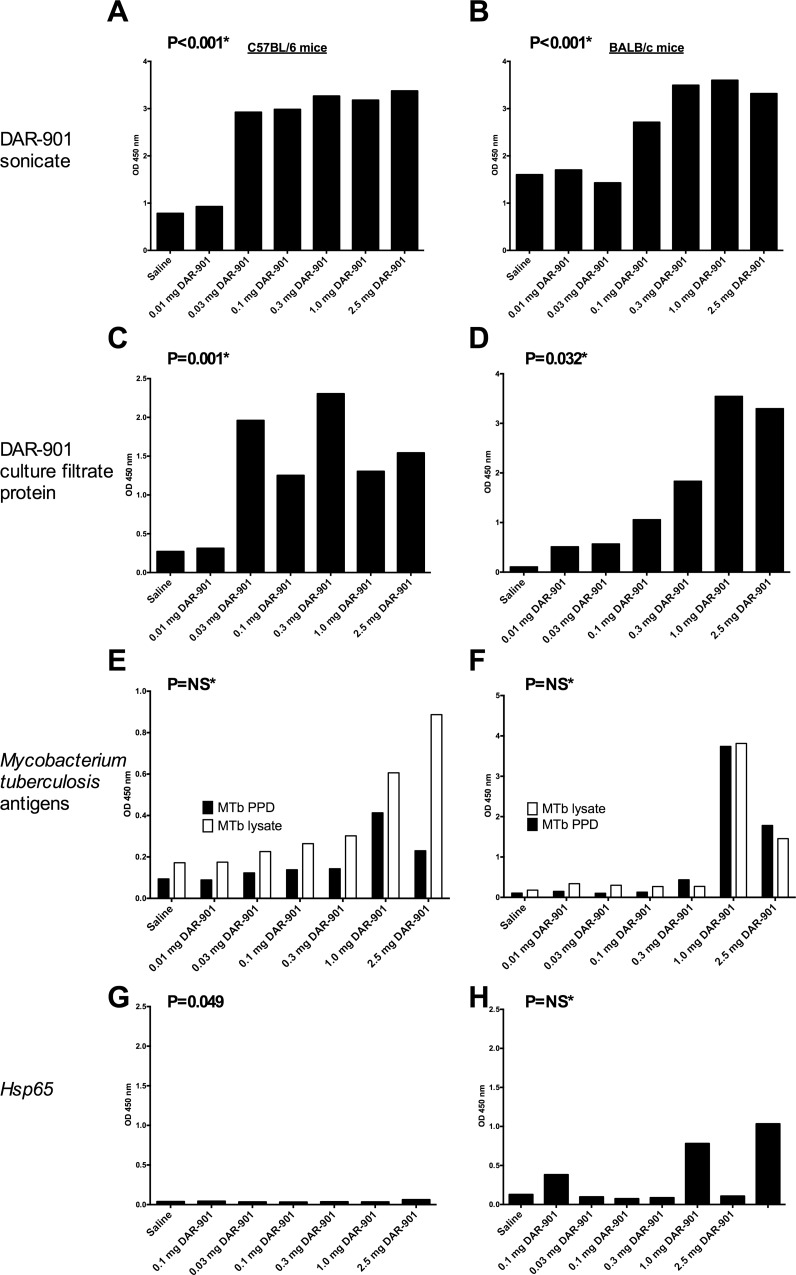
DAR-901 vaccination elicits antibody responses to multiple mycobacterial antigens by enzyme linked immunosorbent assay. Mice were immunized at week 0, 2 and 4 with the indicated dose of DAR-901. At week 2, DAR-901 vaccination enhanced antibody responses to DAR-901 sonicate in C57BL/6 (A) and BALB/c (B) mice, DAR-901 culture filtrate protein in C57BL/6 (C) and BALB/c (D) mice but not to *Mycobacterium tuberculosis* purified protein derivative and lysate in C57BL/6 (E) or BALB/c (F) mice. DAR-901 vaccination enhanced antibody responses to Hsp65 in C57BL/6 (G) but not BALB/c (H) mice, although overall responses were very low magnitude. Serum from 5 mice/dose level was pooled and assayed in duplicate and the bars indicate the mean of two measurements. P values reflect the comparison of all DAR-901 dose levels averaged together to the saline control condition using a student’s t test. MTb, *Mycobacterium tuberculosis*; NS, non-significant; PPD, purified protein derivative

Compared to saline control, DAR-901 vaccination enhanced antibody responses to DAR-901 sonicate (Fig 3A and 3B) and DAR-901 culture filtrate protein in both C57BL/6 and BALB/c mice (Fig 3C and 3D), but not to *M*. *tuberculosis* antigens (Fig 3E and 3F). Antibody responses to mycobacterial Hsp65 were very low magnitude in all conditions but significantly greater at some doses among DAR-901 vaccinees compared to saline controls in C57BL/6 mice but not BALB/c mice (Fig 3G and 3H, respectively).

### Challenge study

Following the second booster vaccination, all 40 DAR-901 mice showed redness and swelling (nodule formation) at the site of injection. In 4 mice (one each receiving 0.1 mg and 2.5 mg, and two receiving 0.3 mg DAR-901) the nodules ruptured, producing open wounds that failed to improve within 24 h after treatment with topical antiseptic. In response to veterinary advice and consistent with the protocol provisions and national regulations, these mice were terminated and the third booster vaccination was removed from the protocol. One mouse from the group with homologous BCG prime and boost was found at daily check approximately four weeks after the boost to appear distressed and in poor health and was also terminated. Post-mortem did not identify any obvious causes for the deterioration which was assessed as unrelated to the vaccination procedure. One mouse in the saline group was found dead approximately 4 weeks post-challenge. This animal had appeared normal on the previous day and no signs of distress had been observed. This event was also assessed as unrelated to the vaccination procedure.

**Fig 4:**
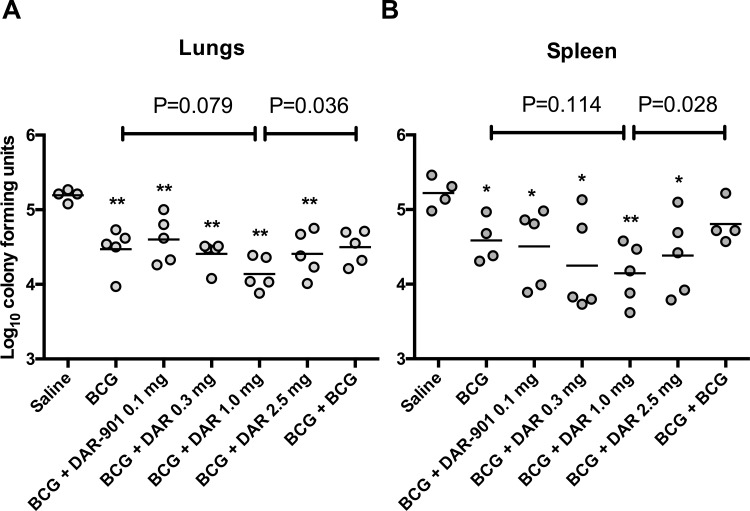
DAR-901 booster vaccination at 1 mg provided greater protection against aerosol challenge with *Mycobacterium tuberculosis* (MTb) in C57BL/6 mice compared to a BCG booster vaccination. Mice were primed with BCG immunization at week zero, and boosted with the indicated immunization at weeks 12 and 14. At study week 20, 5 mice/group were challenged with approximately 15 CFU of Mtb strain H37Rv via the aerosol route. Twelve weeks post-challenge, bacterial burdens in the lungs (A) and spleens (B) of each mouse were enumerated. Growth of MTb in both organs was reduced by immunization with BCG both with or without boosting with DAR-901 at different dose levels. In both tissues, there was a trend toward greater reduction of MTb growth when BCG was followed by DAR-901 up to 1.0 mg dose. Further, in both tissues, BCG-primed animals boosted with DAR-901 at the 1.0 mg dose demonstrated significantly greater suppression of MTb growth than animals given a homologous BCG boost. Each point indicates a single mouse; bars indicate the mean value for that experimental condition. Numerical P values at the top of the figure reflect the results of a student’s t test for the indicated comparison, and are the only comparisons between BCG +/- DAR-901 vaccine conditions that yielded P values < 0.2. * P value for DAR-901 response compared to saline <0.05. ** P value for DAR-901 response compared to saline < 0.01. BCG, bacille Calmette-Guerin

We assessed the growth of MTb in the lungs and spleen of mice primed with BCG with or without booster of DAR-901 or BCG (Fig 4). A single BCG vaccination conferred protection from challenge with MTb (compared to saline, p value in lungs 0.002 and in spleen 0.013). In both lungs and spleen DAR-901 boosting at the 1.0 mg dose resulted in a statistically significant reduction of MTb growth compared to a homologous BCG boost. In addition, there was a trend toward increasing protective efficacy in animals boosted with increasing doses of DAR-901.

## Discussion

SRL172 is the only modern tuberculosis vaccine demonstrated to reduce culture-confirmed tuberculosis in a phase III randomized, double-blind, placebo-controlled, clinical trial [11]. Further development of this vaccine required a more scalable manufacturing process, which has been addressed by the manufacture of DAR-901 from the SRL172 Master Cell Bank via a broth-based method. We now show DAR-901 is immunogenic and, when administered as a booster vaccine for BCG primed animals, enhanced protection against an aerosol tuberculosis challenge compared to a homologous BCG boost.

DAR-901 elicited IFN-γ and antibody responses in mice. Although the immune correlates of vaccine protection from tuberculosis are not known, defects in IFN-γ expression are associated with enhanced susceptibility to mycobacterial infection [14, 15], and we have shown that IFN-γ responses against multiple mycobacterial antigens predict protection from HIV-associated tuberculosis [16, 17]. However, IFN-γ (and other) cytokine responses to tuberculosis antigens failed to correlate with BCG-mediated protection from tuberculosis in a large clinical trial in South Africa [18] and a vaccine that elicited IFN-γ responses to mycobacterial antigens did not confer protection from tuberculosis in a phase IIb human clinical trial [19]. Although SRL172 immunization was associated with reduced laboratory-confirmed tuberculosis among adults with HIV infection [11], and SRL172 elicited IFN-γ responses to mycobacterial antigens [10], IFN-γ responses to mycobacterial antigens did not correlate with vaccine-mediated protection from tuberculosis. Thus the role of IFN-γ responses in vaccine-mediated immune protection from tuberculosis is unclear.

DAR-901 immunization induced antibody responses to DAR-901 sonicate and culture filtrate protein when comparing all dose levels to the saline control. Antibody responses to *Mycobacterium tuberculosis* antigens, by contrast, were not increased when comparing all dose levels together. Given the elicitation of detectable cross-reactive IFN-γ responses after DAR-901 vaccination, the absence of detectable cross-reactive antibody responses does not suggest the absence of cross-reactive antigens although it may hint that the cross-reactive antigens present in DAR-901 are more effective at eliciting IFN-γ responses than antibody responses. Some human and experimental data supports a role for antibody in protection against tuberculosis, although cross-protective antigens were not well-defined, while other studies showed protection may be achieved in the absence of antibody [20–23].

BCG prime followed by two doses of DAR-901 at 1.0 mg dose conferred enhanced protection from tuberculosis challenge compared to mice boosted with BCG. In studies such as this in which immune responses or protection from tuberculosis in a small number of genetically homogeneous animals are assessed, there is real risk of both type I and type II error. We did not correct for multiple comparisons in order to avoid type II error in these pilot studies investigating multiple different dose conditions, and believe our results are plausible in part because they align exactly with prior mouse and human studies. Mouse and other animal models of tuberculosis vaccine efficacy which do not exhibit the latent phase of infection–such as characterizes the vast majority of human infections with *Mycobacterium tuberculosis*–have not yet been able to distinguish between effective and ineffective tuberculosis vaccines in humans [24, 25]. For instance, the tuberculosis booster vaccine candidate MVAg85A protected from tuberculosis after parenteral and intranasal administration in animal models [26–28] but did not protect human infants from tuberculosis when given parenterally in a prime boost trial design [19, 29]. Thus, protective efficacy in animal models should be weighed along with other important considerations when advancing a candidate into clinical development.

Our data build on previous studies showing that heat inactivated whole mycobacterial vaccines are immunogenic and confer protection from *Mycobacterium tuberculosis* challenge in mice [30, 31]. Many tuberculosis vaccine candidates, in fact, are being evaluated in animal models and human clinical trials [3, 32, 33], and, like DAR-901, have exhibited immunogenicity and/or protective efficacy in animal studies.

Only SRL172 has been associated with protection from laboratory-proven human tuberculosis in a phase III randomized, double-blind, placebo-controlled, clinical trial [11]. DAR-901, which was manufactured from the Master Cell Bank of SRL172 using a robust scalable method, offers the safety and polyantigenic immunogenicity of a heat-inactivated, whole-cell product, and thus represents a promising new tuberculosis vaccine candidate. A phase 1 trial of the safety and immunogenicity of DAR-901 in BCG immunized adults with and without HIV infection has been completed in the United States and a Phase 2 prevention of infection trial in BCG-immunized adolescents initiated in Tanzania.

## Supporting Information

S1 Data The GraphPad Prism data file used to generate the figures in this manuscript. (PZF)
